# Medication compliance by cat owners prescribed treatment for home administration

**DOI:** 10.1111/jvim.17298

**Published:** 2025-01-11

**Authors:** Thomas F. Odom, Christopher B. Riley, Jackie Benschop, Kate E. Hill

**Affiliations:** ^1^ School of Veterinary Science Massey University Palmerston North New Zealand; ^2^ Department of Clinical Studies Ontario Veterinary College, University of Guelph Guelph Ontario Canada

**Keywords:** antibiotics, antimicrobials, oral, palatability

## Abstract

**Background:**

Most veterinary literature examining medication compliance has described the phenomenon in dogs. The evidence available regarding factors affecting cat owner medication compliance is limited.

**Objectives:**

Identify and describe factors associated with cat owners' noncompliance with veterinary recommendations for pet medications, as well as client‐reported barriers and aids to administering medications prescribed by primary care veterinarians.

**Subjects:**

Cat owners presenting their animals for veterinary examination and treatment.

**Methods:**

A cross‐sectional survey of cat owners' compliance with veterinary medication recommendations was performed from January 9, 2019, to July 18, 2020. A convenience sample of owners prescribed medication for their pets by veterinarians during or after elective veterinary examination was recruited to respond to questions regarding medication administration experience and compliance. Follow‐up was obtained from owners to determine if the course of medication had been completed. Compliance data were analyzed descriptively, and logistic regression was performed.

**Results:**

Medication noncompliance was recorded for 39% (26/66) of cat owners. A quarter (16/66) reported challenges in administering medication to their pets; the most commonly cited reason was a resistant pet. Oral administration of antibiotics was significantly associated with noncompliance (*P* = .01). Clients with limited pet ownership experience were less likely to be noncompliant (*P* = .04).

**Conclusions and Clinical Importance:**

Clients' inability to medicate their cats PO may have implications for clinical outcomes and antimicrobial stewardship. Alternatives to direct PO administration of solid‐form medications in cats should be considered. Demonstrating administration techniques to all clients may improve compliance and influence clinical outcome.

**Abbreviations**
95% CI: 95% confidence interval
AIC: Akaike information criterion
Est.: coefficient estimate
IQR: interquartile range
MUVTH: Massey University Veterinary Teaching Hospital
OR: odds ratio

## 1 INTRODUCTION

Client compliance with prescribed medication and instructions to treat their pets is a concern in veterinary practice.^1^ Compliance generally refers to the extent to which the human patient follows the prescriber's recommendations, whether correctly self‐administered at the recommended dose or time intervals.^2^ In the case of cat owners, medication compliance is more complex because it requires an understanding of both owner and animal behaviors and their interactions.^3^ Owner compliance is fundamental to successful clinical outcomes for the cat.^4^ For example, as few as 21% of owners treating their epileptic dogs are fully compliant with medication instructions,^5^ and poor compliance with long‐term application of topical medications is reported in cats with glaucoma.^6^ Noncompliance can occur because of cat owners under‐dosing, missing doses, or stopping the medication completely.^7^ Peer‐reviewed literature describing client medication compliance and associated barriers to treating their cats is scarce.^1^, ^8^, ^9^, ^10^, ^11^

Cats present a unique challenge for medication administration. They are, by nature, more discriminating than dogs and, thus, more difficult to medicate.^8^ Less accustomed to restraint than dogs, they are more likely to display fearful behavior or resistance when medicated.^10^ Individual behavior and temperament of the cat, combined with the owner's potential fear of injury, make cats more challenging than dogs to medicate consistently.^10^, ^12^ Clients often report their cat will spit a tablet out, and up to 45% of cats attempt to bite or scratch their owners while being medicated.^11^ Although free‐choice acceptance has been shown to increase compliance,^4^, ^8^ poor palatability of medication can inhibit the treatment regimen's success.^10^, ^12^ Medicating cats may also cause them to avoid interaction with their owners, thus damaging the owner‐cat relationship.^11^, ^13^ The veterinarian's ability to predict which clients will be compliant is unreliable.^14^, ^15^ Nevertheless, the client's suspected ability to successfully medicate their cat may influence the veterinarian's medication selection, potentially affecting patient outcomes.^16^

Few studies have specifically examined the barriers to owner medication compliance, and none have addressed New Zealand cat owners. Of the peer‐reviewed publications investigating medication compliance, most have focused on dogs or cats and dogs collectively, with little to no evaluation of the significance of the contributions of different risk factors for noncompliance.^1^ Most studies performed in cats have been limited to investigations of the palatability or acceptability of PO medications.^4^, ^8^, ^10^, ^17^ Identifying barriers to owners following veterinarians' instructions may help improve owner compliance and patient outcomes for cats.^1^, ^11^, ^18^ We aimed to identify factors associated with the noncompliance of cat owners with veterinary recommendations for dispensed medication administration to their pets, and to describe client‐reported barriers and aids to the administration of medications in a primary care practice in New Zealand.

## 2 MATERIALS AND METHODS

### 2.1 Ethics and data deidentification

Our project was evaluated by peer review as low risk for human participants and was not required to be reviewed by the Human Ethics Committee in New Zealand. It was registered with the Massey University Human Ethics Committee (Human Ethics Notification 4000020081). Data were collected under the low‐risk notification, and informed owner consent was obtained. Data were anonymized immediately after enrollment by providing clients with their animal's patient number to complete the survey.

### 2.2 Study and survey design

A prospective cross‐sectional study of compliance with veterinary medication recommendations by cat owners was performed from January 9, 2019, to July 18, 2020, using a convenience sample of cat owners who were prescribed medication for their pets by veterinarians of the Massey University Veterinary Teaching Hospital (MUVTH) during or after an elective veterinary examination. An online questionnaire was developed using proprietary software (Qualtrics XM, Seattle, Washington; www.qualtrics.com) in 3 parts: owner demographic information, questions relating to the administration of medications to their pet, and identification of challenges associated with medicating their pet (Appendix S1). The survey included questions with binary, categorical, or semiquantitative response options. Questions relating to medication compliance used Likert scales, whereas questions relating to challenges and barriers allowed for multiple selections and free‐text entry. The survey was piloted with 5 cat‐owning staff members employed at MUVTH to refine questions in an iterative process to ensure valid questions.^19^

### 2.3 Inclusion and exclusion criteria

Clients were invited to participate in the study if their cat was seen by the MUVTH Community Practice service veterinarian and had been prescribed any medication (PO or topical) for ≥3 consecutive days. Orally administered medication was defined as any prescribed drug (tablet, capsule, or liquid suspension) administered by mouth to the cat by the client. Topical medication was defined as any medication applied to the skin, eye, or ear of a cat. As part of informed consent, prospective participants were advised that the investigators were studying medication compliance and the aids and barriers they may have experienced in administering medication to their cats. They were further informed that the study findings would be used to educate and assist pet owners in completing treatment instructions and advised that they were free to withdraw from the study at any time. Cats prescribed medication by specialist veterinarians at the practice, those that had their prescribed medication administered in the hospital by staff, or that died or were euthanized between the time of consent and the time of data collection were excluded.

### 2.4 Dissemination of questionnaires

Consenting clients were given a unique URL link to the online survey at the time of the initial visit and medication prescription, as well as their MUVTH patient number, to allow prescriptions to be matched to individual patients without identifying the client to the researchers. Owners were advised to complete the survey after the prescription was provided. Participating clients were contacted by a staff member 2 weeks later, either in person at a reevaluation appointment or by telephone or email after administering a course of medication. The research team member in contact with the client was not involved in the blinded analyses of the results.

### 2.5 Data analysis

Data from the online survey were imported as a spreadsheet CSV file into R statistical software (Version 3.6.1, R Development Core Team 2020, R Foundation for Statistical Computing, Vienna, Austria) for analysis. Entries that did not meet the inclusion criteria and responses that were not sufficiently complete to evaluate outcomes were excluded from further analysis.

Descriptive statistics were determined for all quantitative study variables. The distribution of continuous variables was evaluated for normality using the Shapiro‐Wilk test. The mean and SD were calculated for continuous data that were normally distributed. For data that were not normally distributed, the median and interquartile range were calculated. For the Likert‐scale questions, median and mode are reported.

Univariable logistic regression analyses were performed to assess factors associated with noncompliance and cat medication administration. The binary outcome measure, noncompliance (yes/no), was defined as failing to give the prescribed medication for the duration of the prescription, failing to give the medication at the specified intervals, or failing to give the treatment. Noncompliance was identified by comparing the directions in the prescription with the client's recorded responses. Any deviation from the veterinarian's instruction within the prescription was identified as noncompliance.

Explanatory variables encompassing client, animal, veterinary visit, and medication factors were explored for their contribution to the outcome. An initial univariable logistic regression was performed for each potential explanatory variable, and *P* values were calculated using the Wald test. Each predictor variable returning a *P*‐value <.2 from the univariate modeling was considered for inclusion in a multivariable model. A stepwise backward elimination procedure then was performed whereby predictive values with the least significant *P*‐value were successively removed until all variables in the final model had a Wald's *P*‐value <.05. Basic diagnostic statistics, including fitted and standardized residuals and leverage, were examined for adherence to model assumptions. No influential points were detected. The models were compared using the analysis of variance of deviance function in R, and the model with the lowest Akaike information criterion (AIC) was chosen.^20^ The findings are presented as odds ratios (OR) and confidence intervals (95% CI) for each predictive variable.

## 3 RESULTS

### 3.1 Descriptive data

A convenience sample of 66 respondents met the study criteria. Table 1 summarizes the frequency of owners' responses to a survey on medication administration to their cats presented to the MUVTH. The proportion of surveyed client cat owners noncompliant with medication instructions was 39% (26/66), resulting in an absolute compliance rate of 61% (40/66). The noncompliant cat owners included 50% (1/2) of those required to administer only topical treatment and 38% (24/64) of those required to give medication PO. The latter included 12 owners who gave concurrent topical treatment.

**TABLE 1:** Frequency table of owners' responses to a survey on medication administration to their cats in New Zealand.

Variable Name	Category	Count (%)
Client factors		
Prior experience with pet ownership	First cat	4 (6)
	Multiple cats	15 (22)
	Multiple pets, multiple species	47 (71)
	Total	66 (100)
Prior experience with pet illness	This pet	11 (17)
	Other pets of the same species	15 (23)
	Multiple species	19 (29)
	Training in animal health	14 (21)
	No experience	7 (11)
	Total	66 (100)
Prior experience with medicating pets	No	2 (3)
	Yes, oral medication	35 (53)
	Yes, eye or ear medication	10 (15)
	Yes, all routes	19 (29)
	Total	66 (100)
Client gender	Male	9 (14)
	Female	57 (86)
	I prefer not to answer	0
	Total	66 (100)
Client age group	<30 years old	14 (21)
	31‐40 years old	16 (24)
	41‐50 years old	10 (15)
	51‐60 years old	18 (27)
	>60 years old	8 (12)
	Total	66 (100)
Client ethnic group	NZ European	50 (76)
	Other European	10 (14)
	Māori	3 (5)
	Asian	3 (5)
	Total	66 (100)
Client educational qualification (highest attained)	High school	14 (21)
	University	30 (46)
	Postgraduate	18 (27)
	Other	4 (6)
	Total	66 (100)
Client annual income	<$14 000	9 (14)
	$14 000‐$48 000	12 (18)
	$48 000‐$70 000	10 (15)
	$70 000‐$100 000	3 (5)
	>$100 000	7 (11)
	Missing	25 (38)
	Total	66 (100)
Client disability?	Yes	1 (15)
	No	65 (85)
	I prefer not to answer	0
	Total	66 (100)
Who administered the medication to the cat?	Myself	42 (64)
	Myself plus others	21 (32)
	Other	1 (2)
	Total	66 (100)
Client understanding of the reason the medication was prescribed	Extremely well	45 (68)
	Very well	17 (26)
	Moderately well	4 (6)
	Slightly well	0
	Not well at all	0
	Total	66 (100)
Veterinary visit factors		
The veterinarian spent enough time explaining	Strongly agree	56 (85)
	Somewhat agree	8 (12)
	Neither agree nor disagree	2 (3)
	Somewhat disagree	0 (0)
	Strongly disagree	0 (0)
	Total	66 (100)
The veterinarian explained the reason for the medication well	Strongly agree	57 (87)
	Somewhat agree	6 (9)
	Neither agree nor disagree	3 (5)
	Somewhat disagree	0 (0)
	Strongly disagree	0 (0)
	Total	66 (100)
Who showed the client how to give the medication(s)?	Veterinarian	31 (47)
	Veterinary student	9 (14)
	Nobody	26 (39)
	Total	66 (100)
Medication factors		
Medication class prescribed as reported by the client	Anti‐inflammatory/pain relief (oral)	35 (53)
	Antimicrobial (oral)	23 (35)
	Behavioral (oral)	6 (9)
	Blood pressure (oral)	4 (6)
	Other oral medication	11 (17)
	Topical medication	5 (8)
	Total medications (oral and topical)	84^a^
Frequency of medication	Once a day or every 24 hours	31 (47)
	Twice a day or every 12 hours	25 (38)
	Three times a day or every 8 hours	4 (6)
	Other	6 (9)
	Total	66
Oral medication prescribed.	Yes	64 (97)
	No	2 (3)
	Total	66
How was the oral medication administered?	Directly into the cat's mouth	23 (35)
	With regular cat food	13 (20)
	With a treat	13 (20)
	Combination of methods	17 (25)
	Total	66 (100)
Missed doses of oral medication?	Yes	14 (21)
	No	52 (79)
	Total	100 (100)
Topical medication prescribed?	Yes	14 (21)^b^
	No	52 (79)
	Total	66 (100)
Challenges with topical medication?	Yes	5 (36)
	No	9 (64)
	Total	14 (100)
Missed dose of topical medication?	Yes	7 (50)
	No	7 (50)
	Total	14 (100)
The owner reported challenges giving prescribed medication?	Yes	16 (24)
	No	50 (78)
	Total	66 (100)
Owner‐reported aids in medicating cats	Food	28 (44)
	Training	9 (14)
	Behavioral modification	6 (10)
	Restraint	5 (8)
	Product	8 (13)
	Nothing	7 (11)
	Total	63 (100)

*Note*: Data were available for 66 cats.

^a^ Clients reported that 19/66 (29%) cats received multiple medications.

^b^ Only 2/66 cats received topical treatment alone; the remainder (12/66) received concurrent oral treatments.

Participating owners were most commonly female, aged between 51 and 60 years, New Zealanders of European ethnicity, and educated to a university level. Most reported prior ownership experience of multiple pets of multiple species. Only 11% had no experience managing pet illness, and 20% reported they had training in animal health. Approximately half had prior experience with medicating pets PO, and another 33% reported they had experience with both PO and topical routes of medication administration. Most owners (63%) administered the medication to their cats without assistance (Table 1).

All owners understood the reason for the medication prescription well, and most strongly agreed that the veterinarian had spent sufficient time explaining the reason for the prescription and that the reason for prescribing the medication was explained well. Most clients (61%) were shown how to administer the medication by a veterinarian or veterinary student, but 39% reported that nobody had shown them how to administer the medication (Table 1).

The most common breed of cat presented was domestic short hair (Table S1). There were more females than males. Median weight of these cats was 4.5 kg (interquartile range [IQR], 3.8‐5.1; n = 53), and the median age was 9.6 years (IQR, 3.7‐13.4; n = 53). Almost all (97%; 64/66) cats were prescribed at least 1 PO medication, and 29% were prescribed multiple medications. Medication classes prescribed (as reported by the client) and frequency of administration are presented in Table 1. Only 33% of clients reported giving prescribed PO medication directly into the cat's mouth; most reported administering it with food, a treat, or a combination of these methods. Twenty‐five percent of clients reported challenges giving the prescribed medication. All 16 of these owners indicated, “My pet was resistant to my efforts to medicate him/her” as at least 1 of their reasons for difficulty. Owner‐reported aids in medicating their cats and the frequency with which they were used are presented in Table 1.

Cat owners were asked to report what went well with or assisted medication administration (Table 1). The most common approach was using food to hide the PO medication. In the free text section of the survey, 5 owners described various methods of restraint used, and several described using a particular method to avoid being scratched or bitten. Very few reported the use of a low‐stress handling technique. Twelve percent (8/66) of respondents commented positively on the taste of a specific flavored suspension (Metacam, Boehringer Ingelheim Animal Health NZ, Auckland, NZ) and how the cat's acceptance of that medication markedly aided administration. Several owners commented on cutting or crushing the tablets to aid administration.

### 3.2 Data analyses

Results of the univariate logistic regression and variables selected for consideration in the multivariate model are presented in Table 2. Because of data sparseness, all variables were combined into ≤3 categories. Several continuous variables (eg, age and weight) were categorized into terciles. Several variables (eg, experience of client administering medication) were eliminated before screening because of insufficient variation in data. Other variables (eg, client income) were eliminated before screening because of absent data. Because of covariance and confounding, the formulations of treatments were not included in analyses but are listed in Table S2. The results for variables that failed to meet the inclusion criteria are included in Table S3.

**TABLE 2:** Univariate analyses.

Variable name	Category	Est.^a^	SE^b^	OR^c^	95% CI^d^	*P* ^e^
Weight of cat	<3.9 kg	Ref				
	>3.9 and <4.8 kg	−0.96	0.71	0.38	0.09‐1.49	.17
	>4.8 kg	−0.57	0.69	0.57	0.14‐2.15	.41
Client experience with pet ownership	None	Ref				
	Multiple cats	−0.61	0.77	0.55	0.34‐4.73	.43
	Single cat	−1.85	1.11	0.16	0.39‐5.04	.09
Medication class	No	Ref				
	Anti‐inflammatory	1.37	0.57	3.92	1.33‐12.81	.02
Medication class	No	Ref				
	Antimicrobial	1.76	0.57	5.8	1.97‐18.53	.002
Medication class	No	Ref				
	Other	−1.21	0.64	0.30	0.08‐0.98	.06
Multiple medications	No	Ref				
	Yes	2.33	0.63	10.24	3.17‐38.34	<.001
Medication frequency^f^	Once	Ref				
	Multiple	0.81	0.52	2.24	0.81‐6.38	.12
Pet resisted medication	No	Ref				
	Yes	1.16	0.64	3.18	0.94‐11.83	.07
Medication aid	No	Ref				
	Restraint	1.92	1.16	6.82	0.92‐139.5	.10
Topical medication	No	Ref				
	Yes	0.80	0.62	2.22	0.67‐7.77	.19

*Note*: Data were collected from an online survey on owner compliance with medication administration to cats in New Zealand (n = 66) between 2019 and 2020. Variables with a *P*‐value <.20 were selected for initial inclusion in the multivariate logistic regression model based on univariate logistic regression of associations between medication noncompliance and animal, client, veterinary visit, and medication factors.

^a^ Coefficient estimate.

^b^ Standard error.

^c^ Odds ratio.

^d^ 95% confidence interval.

^e^ *P*‐value for variable.

^f^ Highest medication frequency per client.

Results of the multivariable logistic regression are presented in Table 3. A confounding check was performed to examine the effect of client age. Client age was included in the final model to test the interaction among the remaining variables and no significant effect was found. The results of this confounding check are included in Table S4. In the final multivariable model, client experience with pet ownership and medication with PO antimicrobials were associated with noncompliance.

**TABLE 3:** Results of multivariable logistic regression of associations between medication noncompliance and factors.

Variable name	Category	Est.^a^	SE^b^	OR^c^	95% CI^d^	*P* ^e^
Client experience with pet ownership	None	Ref				
	Multiple cats	−1.57	1.08	0.21	0.021‐1.52	.15
	Single cat	−2.74	1.32	0.06	0.003‐0.66	.04
Medication class	No	Ref				
	Antimicrobial	1.84	0.67	6.27	7.77‐25.83	.01
	Other	−1.84	0.91	0.15	0.02‐0.82	.04
Owner age	<30 years	Ref				
	31‐50 years	−0.03	0.92	0.97	0.016‐6.09	.97
	>50 years	1.17	1.01	3.21	0.45‐25.62	.25

*Note*: Data were collected from an online survey on medication administration to cats in New Zealand (n = 66) between 2019 and 2020.

^a^ Coefficient estimate.

^b^ Standard error.

^c^ Odds ratio.

^d^ 95% confidence interval.

^e^ *P*‐value for variable.

## 4 DISCUSSION

Although 61% of New Zealand cat owners in our primary care practice study complied with medication instructions, a substantial proportion was noncompliant. We aimed to identify factors associated with cat owners' noncompliance with veterinary recommendations for medication administration. Although several candidates for associated factors were found in univariate analyses, only 2 were retained as significant: the administration of PO antimicrobials and pet ownership experience.

Comparative data for cats are scant, but our results compare to 66% compliance reported for an international online survey of 2507 cat owners predominantly located in the United Kingdom, North America, and Europe.^11^ However, compliance among these clients was lower than the 73% medication compliance found for a small survey of 46 cat‐owning clients at 9 Finnish clinics,^10^ and considerably lower than the 93% reported for a multi‐institutional study of 54 cat owners prescribed medication for cardiovascular disease.^12^

The dispensing and PO administration of antimicrobial drugs is important to the completion of ongoing treatment, which involves owners as part of their pet's care team.^7^ Although overall compliance rates with antibiotic administration previously have been measured in dogs,^14^, ^21^, ^22^, ^23^, ^24^ our study statistically identified antimicrobials as a risk factor for noncompliance in cats. Pets that are difficult to dose PO may contribute to poor owner compliance, exacerbating fluctuations in tissue and plasma concentrations of an antimicrobial.^25^ In a survey of New York veterinarians and cat owners, veterinarians thought that 20% of owners could not give medications PO, and 10% of owners indicated their pet was impossible to medicate, influencing veterinary antimicrobial prescribing practices.^26^ These results compare with the 21% of noncompliant owners expected to administer medication PO in our study. These arguments have been used to rationalize using long‐acting parenterally administered antimicrobials such as cefovecin in cats and dogs, in conflict with prudent antimicrobial use recommendations.^25^, ^27^, ^28^ In New Zealand, cefovecin (Covenia, Zoetis New Zealand Limited, Auckland, NZ), classified as a critically important antibiotic, was only registered for use in cats in 2022, after our survey was completed. We suggest that empowering the entire veterinary team to communicate the importance and benefits of the prescribed medication, and antimicrobial stewardship, is essential to develop a sense of priority for clients to administer antimicrobial agents appropriately.^3^, ^28^

The successful PO administration of medication to cats is highly influenced by palatability.^8^, ^10^, ^12^ This supposition was confirmed by owners who remarked on the palatability of a single PO suspension (Metacam, Boehringer Ingelheim Animal Health NZ, Auckland, NZ), and its value in aiding administration. Owners in our study most commonly manipulated their cats' food preferences to mask the flavor or texture or both of medications. Although palatability has long been recognized as a barrier to medication acceptance by cats, most studies have focused on diet, and few have investigated PO antimicrobials.^4^, ^8^, ^29^ Palatable liquid forms of pradofloxacin, doxycycline, and amoxicillin‐clavulanic acid are equally accepted by cats during long‐term treatment for lower urinary tract infections.^30^ However, findings are conflicting regarding owner preferences for liquid or solid formulations.^10^, ^11^ A comparison of tablet and liquid formulations of the same antimicrobial in different physical forms may facilitate a better understanding of the interactions between medication texture and flavor in cats.^17^

In the multivariable model, client experience with pet ownership was associated with noncompliance. The curious protective effect of ownership of a single cat compared to multiple cats or multiple species households on noncompliance is surprising. We hypothesize that first‐time cat owners may be more receptive to veterinary team direction or less affected by multi‐pet household behavioral dynamics.^31^ To our knowledge, limited published data exist on the relationship between owners and multi‐pet households regarding the medical treatment of individual cats.^32^, ^33^

In our study, 39% of cat owners reported that nobody had shown them how to administer their pet's prescribed medication. This finding aligns with those of a recent survey, where 45% of cat owners gave a similar response.^9^ Although the impact of veterinary staff demonstrating medication administration on compliance has not been formally studied, improper techniques in delivering medication PO can decrease the dosage received by the pet, decrease medication bioavailability, or risk injury to both pet and owner.^7^ Interestingly, in our study, no link was found between lack of instruction and noncompliance. Although previous research suggests pet owners may forget this guidance, further analysis of the free‐text responses could offer additional insights.^14^ Notably, many who stated they were not shown how to administer the medication indicated that prior experience or confidence made instructions unnecessary. Therefore, the survey design may have skewed results, because some respondents declined demonstrations voluntarily.

Our study had some limitations. Like most client‐directed studies of medication compliance in cats, sample size was small.^10^, ^12^ Small sample size constrained the number of variables that might reasonably be incorporated into a multivariable model of all possible factors associated with noncompliance. The study design, a blinded client survey, presented additional limitations. Owner self‐reporting in veterinary studies may overestimate compliance compared to other measurement methods.^14^, ^22^ Because enrollment in the study was voluntary, compliant owners may have been more likely to participate, and noncompliant owners underrepresented. Although the survey was offered within 2 weeks of the hospital visit, the retrospective nature of the survey may have increased the risk of owner recall bias for self‐reported data. The lack of an organized patient call‐back routine to remind clients about rechecks after prescriptions may have decreased involvement in the study. Clients not completing the entire survey led to missing data. Finally, individuals with animal experience (eg, veterinary students, nurses, veterinary educators) are overrepresented and this factor may have caused overestimation of compliance when compared with the clientele of other practices.

In conclusion, our results confirm the need for approaches that improve client compliance with veterinary instructions for the medication of their cats. A client's prior experience with pet ownership should be considered when prescribing medication to cats Nevertheless, all clients should be shown how to administer the prescribed medications. Poor compliance with PO antimicrobial medication administration may have consequences for treatment success and antimicrobial stewardship. Ensuring that PO medications prescribed for cats are palatable and safe to administer by their owners before patient discharge may improve compliance in this species.

## CONFLICT OF INTEREST DECLARATION

Authors declare no conflict of interest.

## OFF‐LABEL ANTIMICROBIAL DECLARATION

Authors declare no off‐label use of antimicrobials.

## INSTITUTIONAL ANIMAL CARE AND USE COMMITTEE (IACUC) OR OTHER APPROVAL DECLARATION

This project was evaluated by peer review as low risk for human participants and was not required to be reviewed by a Human Ethics committee in New Zealand. It was registered with the Massey University Human Ethics Committee (Human Ethics Notification 4000020081).

## HUMAN ETHICS APPROVAL DECLARATION

Authors declare human ethics approval was not needed for this study.

## Supporting information


**Appendix S1.** Survey form completed by clients participating in the study of cat owner medication compliance.

**Table S1.** The breed and sex of cats included in a survey on medication administration compliance in New Zealand.

**Table S2.** Oral medication formulation breakdown for drugs prescribed for 64/66 cats in New Zealand.

**Table S3.** Results of univariate analyses for medication compliance for cats in New Zealand.

**Table S4.** Results of multivariate analysis for cat variables with confounding check.
