# Supplementary material for: Medication compliance by cat owners prescribed treatment for home administration
Source: J Vet Intern Med. 2025 Jan 11;39(1):e17298. doi: 10.1111/jvim.17298 (PMC11724197; doi:10.1111/jvim.17298)
Supplement: Supplementary file 2 — Table S1. The breed and sex of cats included in a survey on medication administration compliance in New Zealand. [file JVIM-39-e17298-s003.docx]

**TABLE S1.** The breed and sex of cats included in a survey on medication administration compliance in New Zealand.

| Breed | Male | Female | N (%) |
| --- | --- | --- | --- |
| Domestic short hair | 16 | 22 | 38 (70) |
| Domestic medium hair | 1 | 3 | 4 (8) |
| Burmese | 2 | 1 | 3 (6) |
| Persian | 0 | 2 | 2 (4) |
| Birman | 1 | 0 | 1 (2) |
| British Shorthair | 1 | 0 | 1 (2) |
| Devon Rex | 1 | 0 | 1 (2) |
| Domestic longhair | 0 | 1 | 1 (2) |
| Himalayan | 0 | 1 | 1 (2) |
| Maine Coon | 0 | 1 | 1 (2) |
| Siamese | 1 | 0 | 1 (2) |
| Total^1^ | 24 | 30 | 54 (100) |

^1^Signalment data were provided for 54/66 (82%) cats.
