# Supplementary material for: Medication compliance by cat owners prescribed treatment for home administration
Source: J Vet Intern Med. 2025 Jan 11;39(1):e17298. doi: 10.1111/jvim.17298 (PMC11724197; doi:10.1111/jvim.17298)
Supplement: Supplementary file 3 — Table S2. Oral medication formulation breakdown for drugs prescribed for 64/66 cats in New Zealand. [file JVIM-39-e17298-s004.docx]

**TABLE S2.** Oral medication formulation breakdown for drugs prescribed for 64/66 cats in New Zealand.

| Category | Formulation | Number |
| --- | --- | --- |
| Anti-inflammatory/pain reliever (35) | Tablet/capsule  Liquid | 12  23 |
|  | Total | 35 |
| Antimicrobial (23) | Tablet/capsule  Liquid | 15  8 |
|  | Total | 23 |
| Behavioural (6) | Tablet/capsule  Liquid | 6  0 |
|  | Total | 6 |
| Blood Pressure (4) | Tablet/capsule  Liquid | 4  0 |
|  | Total | 4 |
| Other (11) | Tablet/capsule  Liquid | 8  3 |
|  | Total | 11 |
