# Supplementary material for: Medication compliance by cat owners prescribed treatment for home administration
Source: J Vet Intern Med. 2025 Jan 11;39(1):e17298. doi: 10.1111/jvim.17298 (PMC11724197; doi:10.1111/jvim.17298)
Supplement: Supplementary file 4 — Table S3. Results of univariate analyses for medication compliance for cats in New Zealand. [file JVIM-39-e17298-s005.docx]

**TABLE S3.** Results of univariate analyses for medication compliance for cats in New Zealand.

| Variable Name | Category | Est.^1^ | SE^2^ | OR^3^ | 95%CI^4^ | *p*^5^ |
| --- | --- | --- | --- | --- | --- | --- |
| Breed category | Domestic  Purebred | Ref  0.23 | 0.68 | 1.26 | 0.33-5.01 | .73 |
| Sex of cat | Female  Male | Ref  -0.16 | 0.56 | 0.86 | 0.28-2.57 | .78 |
| Weight of cat | <3.9 kg  >3.9 & <4.8 kg  >4.8 kg | Ref  -0.96  -0.57 | 0.71  0.69 | 0.38  0.57 | 0.09-1.49  0.14-2.15 | .17  .41 |
| Age of cat | <7.1 years  >7.1 & <11.8 years  >11.8 years | Ref  0.22  -0.01 | 0.67  0.70 | 1.25  0.88 | 0.34-4.73  0.22-3.48 | .74  .85 |
| Client experience with pet ownership | None  Multiple cats  Single cat | Ref  -0.61  -1.85 | 0.77  1.11 | 0.55  0.16 | 0.34-4.73  0.39-5.04 | .43  .09 |
| Client experience with pet illness | Health Training  None  All other | Ref  0.30  0.30 | 0.95  0.64 | 1.35  1.35 | 0.20-8.86  0.39-5.04 | .75  .64 |
| Client gender | Female  Male | Ref  -0.84 | 0.86 | 0.43 | 0.06-2.06 | .33 |
| Client age | <30 years  31-50 years  >50 years | Ref  -0.11  0.67 | 0.71  0.69 | 0.90  1.95 | 0.23-3.75  0.52-7.94 | .88  .33 |
| Client education | High school  Postgraduate  University | Ref  -0.22  0.41 | 0.76  0.67 | 0.80  1.50 | 0.18-3.63  0.41-5.94 | .77  .55 |
| Medication class | No  Anti-inflammatory | Ref  1.37 | 0.57 | 3.92 | 1.33-12.81 | .02 |
| Medication class | No  Antimicrobial | Ref  1.76 | 0.57 | 5.8 | 1.97-18.53 | .002 |
| Medication class | No  Other | Ref  -1.21 | 0.64 | 0.30 | 0.08-0.98 | .06 |
| Multiple medications | No  Yes | Ref  2.33 | 0.63 | 10.24 | 3.17-38.34 | <.001 |
| Highest medication frequency per vet | Once  Multiple | Ref  0.03 | 0.53 | 1.03 | 0.36-2.88 | .96 |
| Highest medication frequency as per client | Once  Multiple | Ref  0.81 | 0.52 | 2.24 | 0.81-6.38 | .12 |
| Vet spent enough time | Strongly Agree  All Others | Ref  -0.580 | 0.74 | 0.56 | 0.11-2.25 | .43 |
| How well did vet explain | Strongly Agree  All Others | Ref  0.25 | 0.63 | 1.29 | 0.37-4.43 | .69 |
| Who explained medication instructions | Veterinarian  Nobody  Student | Ref  2.7e^-16^  0.40 | 0.55  0.80 | 1.00  1.50 | 0.35 2.97  0.30-7.51 | 1.00  .61 |
| Pet resisted medication | No  Yes | Ref  1.16 | 0.64 | 3.18 | 0.94-11.83 | .07 |
| Medication aid | No  Food | Ref  -0.55 | 0.57 | 0.57 | 0.18-1.76 | .33 |
| Medication aid | No  Technique/ training | Ref  -0.41 | 0.77 | 0.67 | 0.13-2.90 | .60 |
| Medication aid | No  Behavioral modification | Ref  -0.38 | 0.92 | 0.68 | 0.09-3.89 | .68 |
| Medication aid | No  Restraint | Ref  1.92 | 1.16 | 6.82 | 0.92-139.46 | .10 |
| Medication aid | No  Product | Ref  -0.18 | 0.79 | 0.83 | 0.16-3.85 | .82 |
| Medication aid | No  Nothing | Ref  0.08 | 0.82 | 1.08 | 0.19-5.50 | .92 |
| Oral medication how given | Combo  Directly  Food/Treat | Ref  0.14  -1.02e^-15^ | 0.77  0.76 | 1.15  1.00 | 0.26-5.57  0.23-4.77 | .85  1.00 |
| Topical medication | No  Yes | Ref  0.80 | 0.62 | 2.22 | 0.67-7.77 | .20 |
| Challenge giving topical | No  Yes | Ref  0.18 | 1.13 | 1.20 | 0.13-12.82 | .87 |

^1^ Coefficient estimate; ^2^ standard error; ^3^ odds ratio; ^4^ 95% confidence interval; ^5^ p-value for variable.
