# Supplementary material for: Medication compliance by cat owners prescribed treatment for home administration
Source: J Vet Intern Med. 2025 Jan 11;39(1):e17298. doi: 10.1111/jvim.17298 (PMC11724197; doi:10.1111/jvim.17298)
Supplement: Supplementary file 5 — Table S4. Results of multivariate analysis for cat variables with confounding check. [file JVIM-39-e17298-s001.docx]

**TABLE S4.** Results of multivariate analysis for cat variables with confounding check.

| Variable Name | Category | Est.^1^ | SE^2^ | OR^3^ | 95%CI^4^ | *p*^5^ |
| --- | --- | --- | --- | --- | --- | --- |
| Client experience with pet ownership | None  Multiple cats  Single cat | Ref  -1.57  -2.74 | 1.08  1.32 | 0.21  0.06 | 0.021-1.52  0.003-0.66 | 0.15  0.04 |
| Medication class | No  Antimicrobial | Ref  1.84 | 0.67 | 6.27 | 7.77-25.83 | 0.01 |
| Medication class | No  Other | Ref  -1.84 | 0.91 | 0..15 | 0.02-0.82 | 0.04 |
| Owner Age | <30 years  31-50 years  >50 years | Ref  -0.03  1.17 | 0.92  1.01 | 0.97  3.21 | 0.016-6.09  0.45-25.62 | 0.97  0.25 |

^1^ Coefficient estimate; ^2^ standard error; ^3^ odds ratio; ^4^ 95% confidence interval; ^5^ p-value for variable.
